# Establishment of Norms for Facial Discriminative Sensitivity in Healthy Women Aged 45–60 Years: A Reference Framework

**DOI:** 10.3390/jcm14144884

**Published:** 2025-07-09

**Authors:** François-Régis Sarhan, Thomas Davergne, Christine Couturaud, Sylvie Testelin, Stéphanie Dakpé

**Affiliations:** 1 UR 7516 CHIMERE, Université de Picardie Jules Verne, 80000 Amiens, France; testelin.sylvie@chu-amiens.fr (S.T.); dakpe.stephanie@chu-amiens.fr (S.D.); 2 Physiotherapy School, CHU Amiens-Picardie, 80000 Amiens, France; 3 Maxillo-Surgery Departement, CHU Amiens-Picardie, 80000 Amiens, France

**Keywords:** facial discriminative sensitivity, sensory recovery, two-point discrimination (TPD) test, aging and sensory perception, normative values

## Abstract

**Background/Objectives:** In the context of facial surgery, particularly reconstructive procedures, sensory recovery is a critical yet often underexplored aspect of functional rehabilitation. Sensory-motor recovery can be considered a key marker of integration following reconstructive surgery. Among sensory modalities, discriminative sensitivity is typically the last to recover, making its evaluation particularly relevant. While established norms for hand sensitivity exist in the literature, there is a paucity of data regarding facial sensitivity. The objective of this study was to evaluate the discriminative sensitivity of the face in a population of healthy women aged 45–60 years. **Methods**: A total of 20 healthy women were included between January and March 2013. Participants had no history of facial pathologies or trauma. Discriminative sensitivity was measured using the Disk-Criminator™ device across eight facial zones. A detailed mapping of the tested areas was performed. Data obtained were compared with the existing literature. Statistical analyses included Shapiro–Wilk tests for normality, followed by Student’s *t*-tests for group comparisons. To account for small sample size and verify robustness, non-parametric Mann–Whitney U tests were also performed. Adjustment for multiple comparisons was applied using the Bonferroni correction (adjusted α = 0.0125). **Results**: The mean age of participants was 52.3 years (±4.0 years). Discrimination threshold values ranged from 2.9 to 14.3 mm. Comparison with existing studies showed no significant age-related differences in zone 2R (cheek) and zone 8 (lower lip), suggesting stable sensitivity in these regions across adulthood. However, a significant decline in sensitivity with age was observed only in zone 1R (forehead), with a *p*-value < 0.001 after Bonferroni correction. **Conclusions**: We established a reference framework for cutaneous discriminative sensitivity across eight facial zones. These norms can serve as a baseline for the assessment and monitoring of patients with facial pathologies. Furthermore, our findings contribute to a better understanding of age-related sensory changes.

## 1. Introduction

### 1.1. Background/Rationale

The restoration of facial function through reconstructive surgery heavily relies on an understanding of facial discriminative sensitivity, particularly as it pertains to patient recovery and satisfaction [1,2]. Sensory recovery is a critical yet often underexplored aspect of functional rehabilitation in facial surgery, particularly in reconstructive procedures [3,4]. Sensory-motor recovery can be considered a key marker of integration following surgical intervention [2,4]. Among sensory modalities, discriminative sensitivity—referring to the ability to distinguish between different stimuli on the skin—is typically the last to recover [5,6].

**Figure 1:**
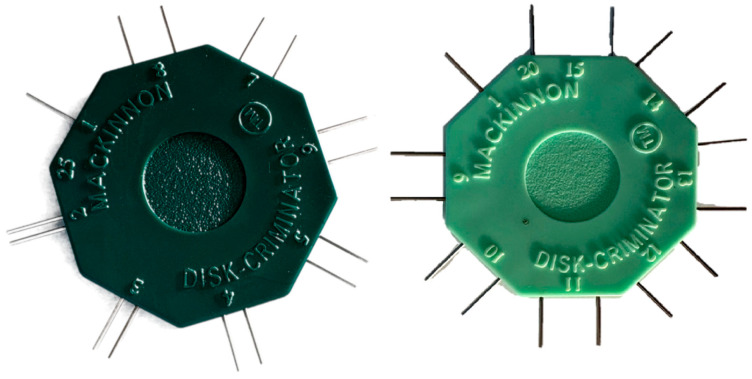
Two-point discrimination device (i.e., “Disk-Criminator™”).

One of the most commonly used methods to assess discriminative sensitivity is the Weber Two-Point Discrimination (TPD) Test [7,8]. This test evaluates the ability to distinguish two closely spaced stimuli as separate points rather than a single touch. It is performed using a device with two sharp points (Figure 1) that are gradually moved closer together until the subject perceives only one point. This technique is widely used in clinical practice to assess tactile perception and has been applied to various anatomical regions, including the hands [7,9] and face [8,10].

While extensive studies have been conducted on sensory thresholds in other regions of the body, such as the hands [11], normative data on facial discriminative sensitivity remains limited [12,13,14,15]. Most available data have been established in young adults [12] or adults younger than 45 years [14,15], which may not accurately reflect sensory characteristics in older populations. Given that cutaneous aging is associated with structural and functional changes, including alterations in mechanoreceptor density, skin elasticity, and neural processing [16,17], it is crucial to determine whether previously established sensory norms remain valid across different age groups or if they evolve with aging. Furthermore, oncologic conditions requiring facial reconstructive surgery predominantly affect individuals aged 45 and older [18], reinforcing the relevance of studying this population. Sensory perception may also vary between men and women, as differences in skin thickness, collagen density, and hormonal influences have been shown to impact mechanoreceptor function and tactile sensitivity [19,20].

### 1.2. Objectives

The primary objective of this study was to establish normative values for facial discriminative sensitivity in healthy women aged 45–60 years. This specific age group was selected as it represents a critical period for facial aging and potential surgical intervention. Previous studies have primarily focused on younger populations [10,11,12,14,15], leaving a gap in knowledge regarding how facial sensory discrimination may change with age.

By assessing sensory discrimination across multiple facial zones, this study aims to determine whether existing normative values from younger cohorts can be extrapolated to older individuals or if adjustments are necessary to account for aging-related changes. As reconstructive surgery continues to evolve, understanding the intricacies of sensory recovery is paramount to optimizing patient outcomes. The reference data generated in this study will contribute to a better understanding of post-surgical sensory rehabilitation and may assist in developing targeted therapeutic strategies for patients experiencing facial sensory deficits.

## 2. Method

### 2.1. Study Design

This study was designed as a cross-sectional observational study aimed at establishing normative values for facial discriminative sensitivity in healthy women aged 45–60 years. The methodology adhered to the STROBE (Strengthening the Reporting of Observational Studies in Epidemiology) guidelines for observational studies [21].

### 2.2. Setting

The study was conducted between January and March 2013 at the CHU Amiens-Picardie, France. Volunteers were recruited from among the hospital staff.

### 2.3. Participants

A total of 20 healthy women aged 45–60 years were recruited for the study. The inclusion and exclusion criteria were defined to ensure a homogeneous sample and to prevent confounding factors related to sensory impairment. Participants were included if they were female, between 45 and 60 years old, and had no history of facial pathology or trauma. To be eligible, participants had to demonstrate sufficient thermal and gross tactile sensitivity to undergo testing.

Participants were excluded if they had prior facial sensory disorders, a history of heavy smoking or alcohol consumption, diabetes, previous surgical interventions or trauma affecting the face, facial pain or chronic headaches, cervical spine disorders with neurological involvement, or ongoing treatment with pain medication, narcotics, neuroleptics, or anxiolytics. Individuals with central nervous system disorders were also excluded.

### 2.4. Variables

The primary outcome variable was facial discriminative sensitivity, which was defined as the smallest distance at which two points could be perceived as distinct. Measurements were recorded in millimeters (mm) across eight predefined facial zones.

### 2.5. Data Sources and Measurement

**Figure 2:**
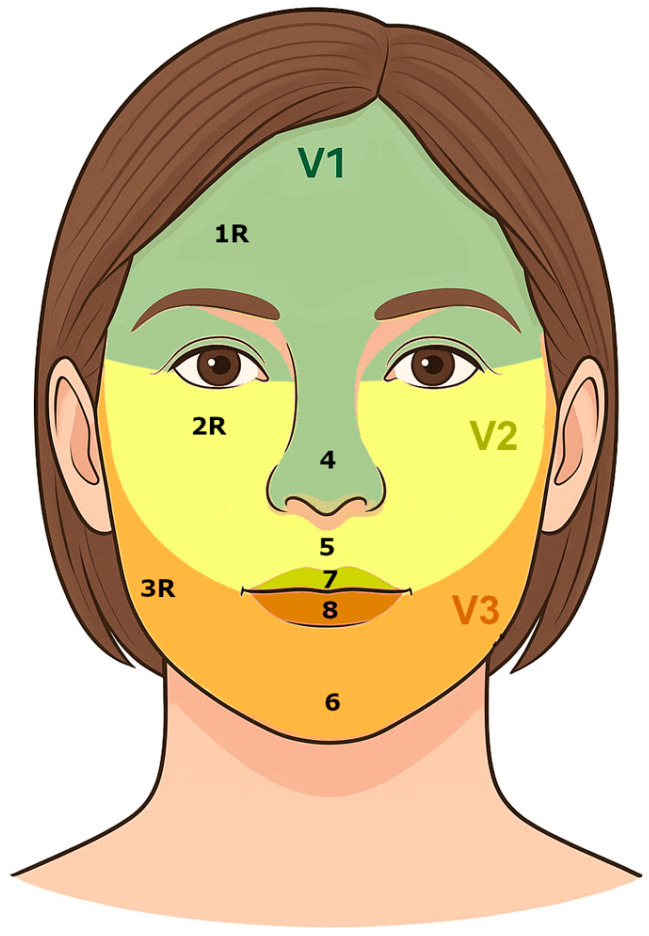
Anatomical localization of the eight facial zones assessed: zone 1R (forehead, V1), zone 2R (cheek, V2), 3R (jaw, V3), 4 (nose), 5 (philtrum), 6 (chin, V3), 7 (upper lip), and 8 (lower lip).

Facial discriminative sensitivity was assessed using the Disk-Criminator™ device (The MacKinnon-Dellon Disk-Criminator-North Coast Medical, Inc., Morgan Hill, CA USA). Testing was performed on eight facial zones covering the three branches of the trigeminal nerve (V) (Figure 2). The zone 1R (forehead) corresponded to the ophthalmic branch V1, the zone 2R (cheek) to the maxillary branch V2, and the zone 3R (jaw) to the mandibular branch V3. Additional zones included the zone 4 (nose), the labial (7–8) and mental regions (6).

For consistency, only the right hemiface was tested, as previous studies have demonstrated no clinically relevant lateral differences in facial sensitivity [12]. Participants were seated comfortably in a quiet room, and all assessments were performed under standardized conditions to ensure reproducibility.

The staircase method, as described by Dellon et al. for trigeminal and peripheral nerve testing [7,8], was used to determine the two-point discrimination threshold. The test began with an initial spacing of 25 mm between the two points of the device, corresponding to the maximal separation allowed by the instrument and ensuring the perception of two distinct points in nearly all cases.

The spacing was progressively reduced, and participants were asked to indicate whether they perceived one or two distinct points. The test was terminated when the participant reported perceiving only one point in two consecutive trials. The final recorded threshold corresponded to the last spacing at which the participant gave two consecutive correct responses to two-point stimulation. This decision rule is derived from standardized procedures used in previous clinical studies [8].

Each stimulus was applied for approximately two seconds. The applied pressure was considered adequate when the skin began to blanch, a visual endpoint commonly used to standardize force in cutaneous sensitivity testing [7]. Participants were instructed to respond “two” if they perceived two distinct points and “one” if they perceived only a single point.

### 2.6. Bias

Several measures were taken to minimize bias. All measurements were conducted by the same trained examiner to reduce inter-rater variability. First, all assessments were conducted by the same trained examiner to reduce inter-rater variability. Importantly, participants were effectively blinded to the stimuli applied, as the tested areas were located on their own face and could not be seen during the procedure. Therefore, participants had no visual cues and based their responses solely on tactile perception, ensuring perceptual objectivity.

Although the examiner was not formally blinded to the stimulus being applied, this limitation is unlikely to have significantly influenced measurement validity in this specific context. The protocol involved a standardized descending staircase approach with strict adherence to procedural neutrality. No verbal or non-verbal cues were given to participants, and responses were binary and participant-driven (“one” or “two” points). Nonetheless, the absence of blinding may have introduced a minor risk of unconscious bias or variability, which should be acknowledged.

Furthermore, the testing environment was carefully standardized: all procedures were conducted in a quiet, temperature-controlled indoor setting to minimize external sensory interference. Participants were given uniform instructions before the test to ensure consistency in task understanding.

### 2.7. Study Size

An a priori power analysis was performed using GPower 3.1.9.7. Based on a paired design, an alpha level of 0.05, a power of 0.80, and an expected effect size of 3.0 mm, as suggested by previous studies on minimal detectable differences in two-point discrimination (e.g., Novak et al. [15], SD = 2.8 mm for zone 1R (forehead)), the minimum required sample size was estimated at 18 participants. Including 20 participants provided sufficient power to detect meaningful differences in facial sensitivity.

Missing data were handled using listwise deletion for analyses involving zones 2R (cheek), 4 (nose), and 6 (chin), where complete data were not available for all participants. This method was appropriate given the small number of missing values and the lack of evidence for systematic bias.

### 2.8. Quantitative Variables

Facial discriminative sensitivity was recorded in millimeters, and data were collected for each tested facial zone. The study analyzed inter-individual variability in sensory perception.

### 2.9. Statistical Methods

The normality of the data was verified using the Shapiro–Wilk test. Descriptive statistics were used to analyze the results, with mean values and standard deviations calculated for each facial zone. Reference values were determined using a 95% confidence interval. In addition, the coefficient of variation was calculated to assess the relative dispersion of the data.

Comparisons with existing reference values from younger populations were performed to assess potential age-related changes in discriminative sensitivity. To this end, we compared the sample sizes, means, and standard deviations of the identified studies with our data using unpaired Student’s *t*-tests. In addition, to validate the robustness of our results given the small sample size, we conducted non-parametric comparisons using the Mann–Whitney U test as a sensitivity analysis. Given the multiple comparisons conducted across four facial zones, a Bonferroni correction was applied to adjust the significance threshold, setting it at α = 0.0125 (0.05/4). All statistical analyses were conducted using JASP software (JASP Team, 2024; Version 0.19.3).

### 2.10. Ethical Considerations

The study was conducted in accordance with the Declaration of Helsinki and adhered to ethical principles for medical research involving human participants. Written informed consent was obtained from all participants before enrollment. The study followed institutional guidelines for research ethics applicable at the time of data collection (2013) and complied with standard regulatory practices for observational studies.

## 3. Results

### 3.1. Sample Characteristics

A total of 20 female participants, aged 47 to 60 years, were included in the study. No participants were excluded after screening, as all volunteers met the inclusion criteria and reported no neurological disorders, facial trauma, or systemic conditions affecting cutaneous sensitivity.

The mean age was 52.3 years (±4.0). Regarding the measurements, 20 values were recorded for points 1R (forehead), 3R (jaw), 5, 7, and 8. However, only 19 measurements were available for points 2R (cheek), 4, and 6 due to a data entry error by the operator. No shared demographic characteristics or procedural irregularities were found for these cases, supporting the assumption that the missing values were missing completely at random (MCAR).

The Shapiro–Wilk test confirmed a normal distribution of all variables, allowing their description using means and standard deviations and enabling the use of parametric tests.

### 3.2. Main Results

**Figure 3:**
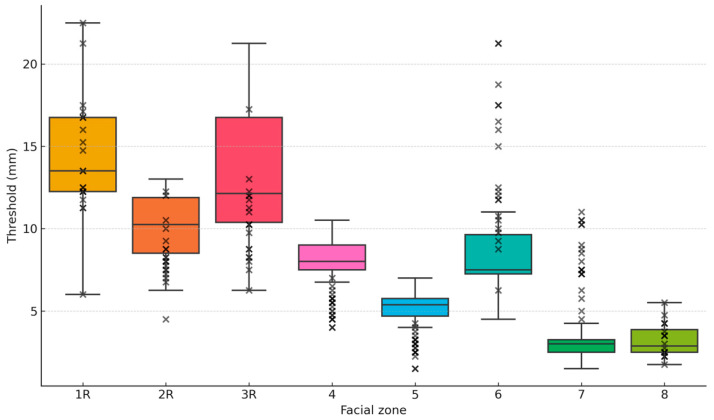
Distribution of two-point discrimination thresholds across the eight facial zones in 20 healthy women aged 45–60 years. Facial zones are presented in the following order: zones 1R (forehead), 2R (cheek), 3R (jaw), 4 (nose), 5 (philtrum), 6 (chin), 7 (upper lip), and 8 (lower lip). Each boxplot displays median, interquartile range, and individual data points. Statistical comparisons with previously published normative data (Novak et al., 1993 [15]) were performed using paired Student’s *t-*tests; significance level was adjusted using Bonferroni correction (α = 0.0125).

The results are summarized in Table 1. Mean discrimination thresholds varied across facial regions, ranging from 2.9 mm in zone 7 (upper lip), the most sensitive area, to 14.3 mm in zone 1R (forehead), the least sensitive (Figure 3). The 95% confidence intervals were relatively narrow, indicating high precision in the estimates. Zones 1R forehead (14.3 mm) and 3R (jaw) (13.5 mm) showed the lowest sensitivity, whereas zones 7 (upper lip) (2.9 mm) and 8 (3.2 mm) exhibited the highest sensitivity.

### 3.3. Inter-Individual Variability

The standard deviation ranged from 0.7 mm in zone 7 (upper lip) to 4.3 mm in zone 3R (jaw), indicating that some facial regions exhibit greater absolute variability than others. However, when accounting for the mean values, the coefficients of variation (0.2–0.3) suggest that the relative variability remains moderate across participants.

### 3.4. Comparison with the Literature Data

**Figure 4:**
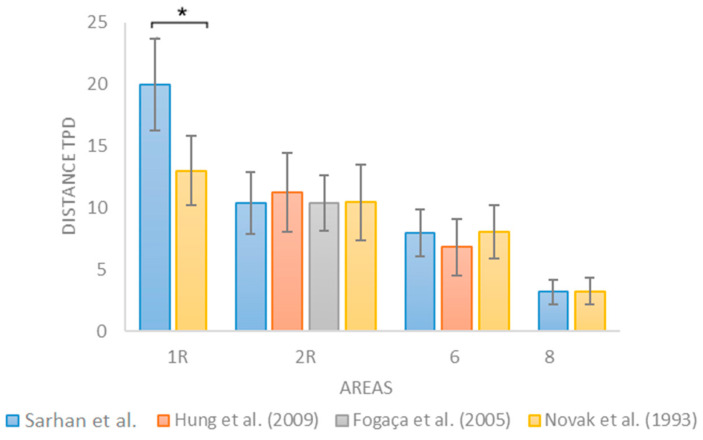
Comparison of two-point discrimination thresholds from the current study and three previously published datasets for zones 1R (forehead), 2R (cheek), 6 (chin), and 8 (lower lip). Sample sizes range from n = 19 to 20 depending on zone completeness. A significant difference (*) was observed in zone 1R (forehead) compared to Novak et al. (1993) [15] (*p* = 5.46 × 10^−8^) [12,14].

Women’s mean values reported by previous studies (Hung et al., 2009 [12]; Fogaça et al., 2005 [14]; Novak et al., 1993 [15]) were compared across common anatomical areas tested (1R, 2R, 6, and 8) (Figure 4).

A highly significant difference was observed in zone 1R (forehead) (Mean = 20.0 ± 3.70) when comparing our cohort to the study by Novak et al. [15] (Mean = 13.0 ± 2.79), with a *p*-value of 5.46 × 10^−8^ using Student’s *t*-test. In area 6 (chin), a statistically significant difference was found with the study by Hung et al. [12] (Mean = 6.81 ± 2.26), with *p* = 0.031. However, after applying a Bonferroni correction for multiple comparisons (adjusted α = 0.0125 for 4 comparisons), only the difference in zone 1R (forehead) remained statistically significant. The difference in zone 6 did not survive correction, suggesting that it may reflect random variation rather than a true age-related effect.

In contrast, no statistically significant differences were observed in areas 2R (cheek) and 8 (lower lip) when compared to values from Hung et al. [12] (2R: *p* = 0.233), Fogaça et al. [14] (2R: *p* = 0.978), and Novak et al. [15] (2R: *p* = 0.954; 8: *p* = 0.902).

As a sensitivity analysis, we performed Mann–Whitney U tests for the same comparisons to account for the limited sample size and potential deviations from normality. The results of the non-parametric tests were consistent with the parametric analysis. Specifically, the differences observed in zones 1R and 6 remained statistically significant (*p* = 1.03 × 10^−6^ and *p* = 0.036, respectively), while comparisons in zones 2R (cheek) and 8 (lower lip) remained non-significant (*p* = 0.883 and *p* = 0.866, respectively). As with the parametric analysis, only the difference in zone 1R (forehead) remained significant after Bonferroni correction (adjusted α = 0.0125).

To further support clinical interpretation, we calculated Cohen’s d for the comparisons in zone 1R (forehead). The difference observed in zone 1R (forehead) between our cohort and Novak et al. (1993) [15] yielded a large effect size (d = 2.11), confirming a substantial age-related decline in discriminative sensitivity.

These findings reinforce the robustness of our results and suggest that zone 1R (forehead) sensitivity declines with age, while zone 2R (cheek) and lip sensitivity remain relatively stable.

## 4. Discussion

### 4.1. Interpretation of Results

Facial discriminative sensitivity plays a crucial role in sensorimotor function and overall facial perception, particularly in the context of reconstructive surgery and neurological assessment. Understanding the variability in sensory thresholds across different facial regions is essential for establishing clinically relevant reference values.

**Figure 5:**
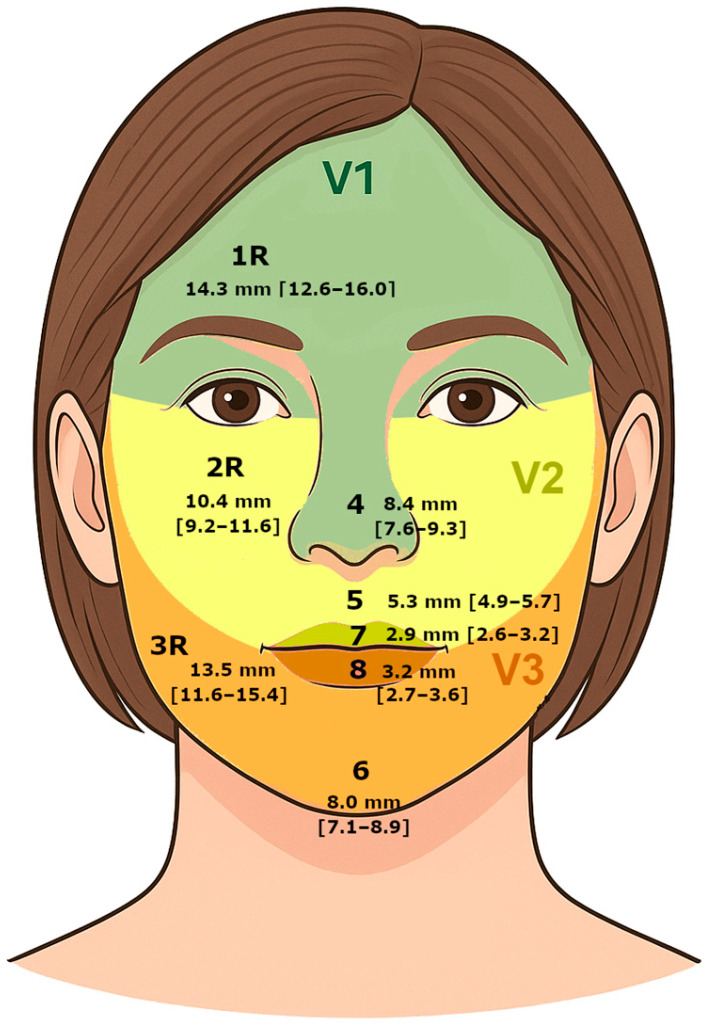
Standardized facial map of two-point discrimination thresholds with 95% confidence intervals. This map illustrates the mean discriminative thresholds (in mm) and corresponding 95% confidence intervals for each tested facial region in women aged 45–60 years, providing normative reference values for clinical assessment.

In this study, we observed notable differences in discrimination thresholds across facial zones, reinforcing the widespread idea that sensitivity is not uniformly distributed. The lowest discrimination thresholds, indicating higher tactile sensitivity, were found on labial zones 7 (upper lip) (2.9 mm) and 8 (lower lip) (3.2 mm). In contrast, the highest thresholds, reflecting lower tactile sensitivity, were recorded in zones 1R (forehead) (14.3 mm) and 3R (jaw) (13.5 mm) (Figure 5).

From a physiological perspective, one hypothesis could be that these differences are explained by the distribution of mechanoreceptors in the facial skin. Regions with higher receptor density, such as those surrounding the lips and zone 2R (cheek), exhibit greater sensitivity, whereas bony regions like the zone 1R (forehead) and mandible (zone 6) tend to have higher discrimination thresholds due to a lower density of sensory receptors. This could be supported by findings indicating that the central part of the face (V2 region) has an estimated innervation density of 66 units/cm^2^, while the forehead, eyes, and zone 4 (nose) (V1 region) have a lower density of 48 units/cm^2^ [22]. This interpretation should be confirmed by future neurohistological investigations.

From a functional perspective, the high tactile sensitivity of the lips can be compared to that of the fingertips. Studies have reported two-point discrimination thresholds of approximately 2 mm for the lips, which closely resemble the 2–3 mm thresholds found in the fingertips [7,9]. This similarity highlights the critical role of fine tactile discrimination in both regions for executing delicate and precise tasks. In contrast, areas such as the forehead and mandible exhibit higher discrimination thresholds, reflecting a lower density of sensory receptors and reduced tactile sensitivity.

### 4.2. Reliability of Measurements and Data Robustness

The narrow confidence intervals and normal distribution of the data indicate a high level of reliability in the results. The use of the Disk-Criminator™ appears to provide precise and reproducible measurements, reinforcing the validity of the findings. These results suggest that the method used in this study (use of the Disk-Criminator™ and descending staircase method) can serve as a reliable tool for assessing facial sensitivity in both clinical and research settings.

### 4.3. Influence of Age

The mean age of participants varied across different studies assessing facial discriminative sensitivity. Hung et al. [12] reported a mean age of 24 (range 15–35) years, Fogaça et al. [14] included participants with a mean age of 36 ± 13 years, and Novak et al. [15] had a sample with a mean age of 40 ± 13 years. In comparison, the present study investigated an older population with a mean age of 52.3 ± 4 years, allowing for an assessment of potential age-related differences.

No significant age-related differences were observed for zone 2R (cheek) and zone 8 (lower lip), suggesting that tactile sensitivity in these areas remains relatively stable across adulthood. For zone 6 (chin), a difference was initially found when comparing our results with those of Hung et al. (2009) [12]; however, this difference did not reach significance after correction for multiple comparisons and was not observed when compared with Novak et al. (1993) [15]. These findings suggest that sensitivity in the chin area may remain stable beyond the age of 30, with inter-study variations potentially attributable to methodological differences or statistical variability rather than true age-dependent changes.

Although no standardized minimum clinically important difference (MCID) has been established for two-point discrimination thresholds in the facial region, the observed difference of 7 mm in zone 1R (forehead) corresponds to seven incremental graduations on the Disk-Criminator™ device, which may suggest a perceptible change from a clinical perspective.

Similar findings have been reported for the hand [23,24], where less sensitive areas (palm) are more affected by aging than highly sensitive areas (finger pulp), which tend to remain preserved. This pattern appears to extend to the face, with labial sensitivity being maintained, while discriminative sensitivity of the forehead declines with age.

The normative values established in this study offer a useful reference for postoperative sensory evaluation following facial reconstructive surgery. They allow clinicians to assess whether the recovery of facial tactile sensitivity is proceeding within expected parameters and to identify cases where reinnervation may be incomplete or deviating from typical recovery patterns. These data are particularly valuable for supporting rehabilitation strategies aimed at restoring discriminative function, especially in functionally critical regions such as the lips.

In long-term follow-up situations, such as those involving secondary reconstructions or progressive nerve recovery, it becomes essential to consider the patient’s age in interpreting sensory outcomes. The natural decline in facial sensitivity beyond the age of 45 must be accounted for to distinguish normal aging effects from residual postoperative hypoesthesia. This consideration is even more important in contexts where postoperative surveillance extends over several years, as is often the case in complex reconstructive procedures.

Moreover, the relevance of these reference values extends to innovative surgical interventions such as facial allotransplantation, which require lifelong sensory monitoring. In such settings, the ability to situate a patient’s recovery within an established normative framework enhances the precision of clinical evaluation and supports evidence-based decision-making throughout the rehabilitation process.

Finally, although this study was restricted to women to minimize variability, future investigations should aim to establish sex-specific norms to ensure the applicability of these findings to the broader population.

### 4.4. Study Limitations

One limitation of this study is the relatively small sample size, although the population was homogeneous, allowing for consistent comparisons within the targeted age group. Minor data omissions were observed in three zones. As these omissions were randomly distributed and unrelated to participant characteristics or procedural factors, they were managed using listwise deletion. This approach, although justified in this context, highlights the importance of stringent data monitoring procedures in future studies.

The number of facial zones analyzed in relation to age was also limited, constrained by the availability of comparable data in the existing literature. Expanding the analysis to additional regions could provide a more comprehensive understanding of age-related sensory changes. In particular, the zone 6 (chin) warrants further investigation due to the contradictory findings observed across studies.

From a methodological perspective, the absence of test–retest reliability assessment represents a limitation. Although all measurements were performed by the same trained examiner to minimize variability, future studies should include repeated measurements on a subset of participants to establish intra-examiner consistency and the stability of the threshold estimates.

Additionally, while pressure was standardized visually based on skin blanching, as commonly used in similar protocols [7], this approach introduces a degree of subjective variability that could influence results. Previous studies using comparable manual devices have reported intra-rater variability in threshold measurements ranging from 0.5 to 2.0 mm depending on the tested region and methodological rigor. While our use of a descending staircase method mitigates some of this variability, we acknowledge that examiner-dependent pressure inconsistencies may have contributed to measurement dispersion, particularly in zones with lower mechanoreceptor density such as the forehead or chin. Quantitative tools such as calibrated monofilaments or pressure sensors could improve reproducibility in future research. Future research should aim to establish minimum clinically important differences (MCID) specific to each facial region to enhance the clinical interpretability of sensory assessment results.

Finally, further studies should explore the influence of additional factors known to affect cutaneous sensitivity, such as tobacco use, diabetes, and prolonged exposure to extreme climatic conditions. These variables may contribute to inter-individual variability and should be considered in future investigations to refine our understanding of facial sensory aging.

## 5. Conclusions

This study establishes reference values for facial discriminative sensitivity in women aged 45–60 years, filling a critical gap in the literature. Our findings reveal an age-related decline in forehead sensitivity, while labial sensitivity appears preserved. These normative data provide a valuable baseline for clinicians assessing facial sensory function, particularly in the context of facial nerve injury, reconstructive surgery, or facial transplantation. Clinicians may use these thresholds to detect deviations from age-matched norms and monitor recovery over time. Future research should investigate how additional factors, such as smoking, diabetes, or chronic sun exposure, might modulate facial sensory aging and further refine clinical assessment tools.

## Figures and Tables

**Table 1:** Discriminative sensitivity data (TPD).

Area	1R	2R	3R	4	5	6	7	8
n	20	19	20	19	20	19	20	20
Mean TPD (SD)	14.3 (±3.7)	10.4 (±2.5)	13.5 (±4.3)	8.4 (±1.8)	5.3 (±0.8)	8.0 (±1.9)	2.9 (±0.7)	3.2 (±1.0)
95% CI Mean	[12.6–16.1]	[9.2–11.6]	[11.5–15.5]	[7.6–9.3]	[4.9–5.7]	[7.1–8.9]	[2.6–3.2]	[2.7–3.7]
Coefficient of variation	0.3	0.2	0.3	0.2	0.2	0.2	0.2	0.3
*p*-value of Shapiro–Wilk	0.30	0.20	0.30	0.20	0.80	0.40	0.60	0.10

Note: The number of participants (n) varies between 19 and 20 across facial zones due to isolated instances of missing or invalid measurements, such as inconsistent participant responses or procedural errors. These data points were excluded using listwise deletion to preserve data integrity.

## Data Availability

The original contributions presented in this study are included in the article. Further inquiries can be directed to the corresponding author.
